# Inconsistent Effects of Iron-Folic Acid and/or Zinc Supplementation on the Cognitive Development of Infants

**DOI:** 10.3329/jhpn.v29i6.9896

**Published:** 2011-12

**Authors:** Emily H. Siegel, Katarzyna Kordas, Rebecca J. Stoltzfus, Joanne Katz, Subarna K. Khatry, Steven C. LeClerq, James M. Tielsch

**Affiliations:** ^1^Department of International Health, Johns Hopkins Bloomberg School of Public Health, Baltimore, MD, USA; ^2^Department of Nutritional Sciences, Pennsylvania State University, University Park, PA, USA; ^3^Division of Nutritional Sciences, Cornell University, Ithaca, NY, USA; ^4^Nepal Nutrition Project–Sarlahi, Nepal Netra Jyoti Sangh, Kathmandu, Nepal

**Keywords:** Cognition, Cognitive development, Folic acid, Infant, Information processing, Iron, Micronutrients, Zinc, Nepal

## Abstract

Despite concerns over the neurocognitive effects of micronutrient deficiencies in infancy, few studies have examined the effects of micronutrient supplementation on specific cognitive indicators. This study investigated, in 2002, the effects of iron-folic acid and/or zinc supplementation on the results of Fagan Test of Infant Intelligence (FTII) and the A-not-B Task of executive functioning among 367 Nepali infants living in Sarlahi district. Infants were enrolled in a cluster-randomized, placebo-controlled clinical trial of daily supplementation with 5 mg of zinc, 6.25 mg of iron with 25 µg of folic acid, or zinc-iron-folic acid, or placebo. These were tested on both the tasks using five indicators of information processing: preference for novelty (FTII), fixation duration (FTII), accelerated performance (≥85% correct; A-not-B), deteriorated performance (<75% correct and >1 error on repeat-following-correct trails; A-not-B), and the A-not-B error (A-not-B). At 39 and 52 weeks, 247 and 333 infants respectively attempted the cognitive tests; 213 made an attempt to solve both the tests. The likelihood of females completing the A-not-B Task was lower compared to males when cluster randomization was controlled [odds ratio=0.67; 95% confidence interval 0.46-0.97; p<0.05]. All of the five cognitive outcomes were modelled in linear and logistic regression. The results were not consistent across either the testing sessions or the information-processing indicators. Neither the combined nor the individual micronutrient supplements improved the performance on the FTII or the A-not-B Task (p>0.05). These findings suggest that broader interventions (both in terms of scope and duration) are needed for infants who face many biological and social stressors.

## INTRODUCTION

In recent years, the micronutrients—iron and zinc—have received increased attention due to their role in facilitating the growth and development of children. Beginning at six months, children require amounts of zinc and iron that cannot be met by breastmilk alone (1). Children who are fed a predominately plant-based diet often have difficulty in obtaining iron and zinc in a sufficient quantity because the nutrients in plant-based foods, such as green-leafy vegetables (iron) and grains (zinc), are not easily absorbed (2). In Nepal, serum concentrations of zinc have revealed high levels of zinc deficiency (3), and iron-deficiency anaemia (IDA) was found in 48% of children aged less than two years (4).

Both iron and zinc are needed for brain function and are found in areas of the brain that are responsible for higher intellectual functioning (5-8). Iron is a co-factor in enzymes involved in neurotransmitter synthesis and is required for myelination, which ensures proper neuronal signal conduction (5). Zinc is one of the most ubiquitous metals in the brain and is present in many enzymes involved in brain growth, proteins for neurotransmission, and in neurotransmitters used for brain memory function (6,8). The presence and the location of iron and zinc in the brain, together with evidence on their role in neurotransmission, indicate that both metals are important for optimal neural function and cognition.

In turn, deficiencies in iron and zinc are associated with decreased cognitive functioning. Evidence from iron-repletion and supplementation trials demonstrates that children with IDA perform worse than children without IDA on developmental indices—both mental (9-12) and motor (13). Mixed results were found in zinc-supplementation trials among children aged less than two years. Among the studies that examined the traditional sensorimotor outcomes (e.g. Bayley and Griffiths Scales), two revealed differences (14,15) and two found no differences (16,17) between zinc-supplemented and unsupplemented infants. One of these studies discovered that zinc-supplemented infants performed better (14) whereas another found that they did worse (15). In studies that examined the effect of zinc supplementation on different outcomes, including activity level, behaviour, and posture, each found that zinc-supplemented children performed better than unsupplemented children (16,18,19). Selection of an appropriate test is a key to understanding how cognition develops in the young child—both in conditions of optimal nutritional status and when micronutrient deficiencies occur.

Cognition is a complex construct not fully captured by general tests of sensorimotor development [i.e. the Bayley Scales of Infant Development (20)] that are increasingly recognized as having poor construct and predictive validity in children aged less than two years (21,22). On the other hand, specific tests of the infant's information processing (attention, recall, and executive function) are predictive of performance on language-based intelligence tests in older children and adolescents (23-25) but have had a limited use in micronutrient studies focused on infants’ cognition.

We examined the effects of supplementation with iron-folic acid and/or zinc on the information processing of 39-and 52-week-old Nepali children using a randomized placebo-controlled design. We specifically investigated how rural Nepali children perform on information-processing tests, and whether supplementation with iron-folic acid and/or zinc results in better scores on these measures.

## MATERIALS AND METHODS

### Study subjects

The study was conducted on a sub-sample of children drawn from a large community-based trial of daily supplementation of iron-folic acid and zinc designed to investigate the effects of daily supplementation with iron-folic acid and zinc on mortality (3,26). Families were identified as eligible for the large trial from a census conducted by the study personnel during December 2000–March 2001. Children were enrolled in the trial during October-December 2001 following the caregiver's verbal consent. For the present study, a single village development committee (VDC) in Sarlahi district in south central Nepal was selected to investigate the effects of iron-folic acid and zinc supplementation on the developmental outcomes. The religious and caste groups represented in this sub-sample included: Brahmins, Chhetris, Vaiyshas, Shudras, and Muslims.

**Fig. 1.**
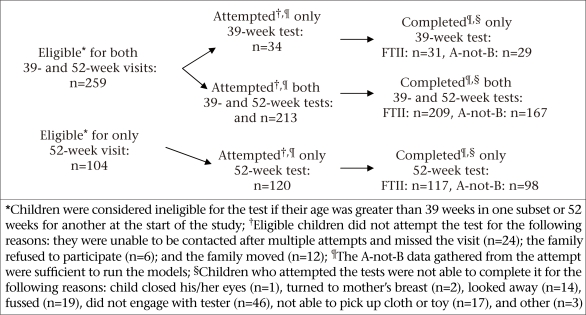
Eligibility and attrition of participating children

**Fig. 2.**
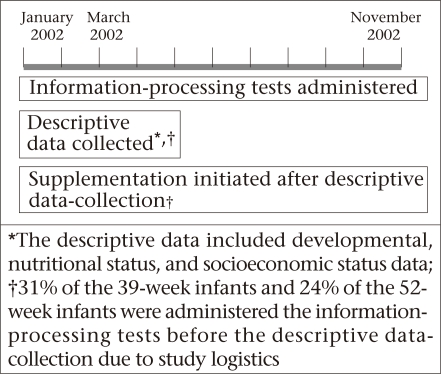
Timeline of study activities

Developmental, nutritional status, and socioeconomic measurements for the child development sub-study were collected during January-March 2002. A sub-set of children enrolled in the child development sub-study (eligibility was determined based on age) was invited to participate in a cognitive-testing protocol when they turned 39 and 52 weeks of age between January and November 2002. These age criteria are dictated by the Fagan Test of Infant Intelligence (FTII)—one of the measures used. After verbal consent from caregivers, 259 children were eligible to receive both the tests, and 104 children, older than 39 weeks at the study launch, were eligible to receive only the 52-week test (Fig. 1). Children whose ages were within the test window at the beginning of the study received the information-processing measures before the developmental, nutritional status, and socioeconomic measurements (Fig. 2).

The study was approved by the Johns Hopkins Bloomberg School of Public Health and the Nepal Health Research Council and is registered at Clinicaltrials.gov (NCT #00109551).

### Assessment of cognitive development, nutritional status, and sociodemographics

Cognitive development was evaluated using the information-processing measures that were part of the FTII (27) and the A-not-B Task (28,29). Children were scheduled for the FTII and the A-not-B Task at the same time during a two-week window around 39 and 52 weeks, assuming term delivery. Only children aged 53 weeks or less at baseline were eligible for these assessments. The tests were administered in mid-morning in the field office—the A-not-B Task before the FTII. Three local fieldworkers achieved at least 85% on inter-rater reliability after being trained to conduct the tests.

The FTII, a paired-comparison test, measures the span of time an infant views a familiar stimulus, identifies it as familiar, and shifts his attention to a novel stimulus (27,30). The FTII generates the preference for novelty score—time spent looking at the novel stimuli divided by the time spent looking at both novel and familiar stimuli, a computation that the program calculates automatically. Another measure—fixation duration—was defined as time spent attending to the stimuli and was indicative of information-processing time. Fixation duration was calculated by dividing the sum of the time spent looking at the left stimulus by the sum of time spent looking at the right stimulus. These data are automatically generated by the FTII program. A faster processing time is indicative of more advanced development.

The A-not-B Task measures the ability of an infant to recognize objects as being permanent and separate from the child (29). The concept of object permanence was described by Piaget as being one of the infant's most important accomplishments (30). In the A-not-B Task, a toy is hidden in one of two wells of the test apparatus, and the child is tasked to find it. To ensure that the challenge is age-appropriate, a time-delay may be imposed by the tester between hiding the toy and allowing the child to reach for it. In this study, the testers administered 1-3-second delay at all ages that increased by two seconds after the first three correct trials for 3-5 seconds.

Two types of trials are administered in this task: one in which the hiding location remains the same as on the previous trial and one in which the hiding location reverses (reversal trial). The A-not-B error occurs when the child fails to look for the toy in the new hiding place on a reversal trial and instead looks for the toy in the previous location. It has been proposed that the error reveals the child's inability to inhibit a previously-rewarded search and/or remember the new location (31).

The study children were marked as having made the A-not-B error if the following conditions were present: (a) no more than one error on a repeat-following-correct trial (the side of hiding remains the same, and the child was correct on the previous trial); (b) at least one error on a reversal trial; and (c) either an error on one more reversal trial or an error on the trial immediately following the reversal error. Children were classified as having accurate performance when they were able to locate the toy on at least 85% of the trials. They experienced deteriorated performance when they found the toy on less than 75% of the trials and had more than one error on repeat-following-correct trials (31). A repeat-following-correct trial occurred when the side of hiding remained the same, and the subject was correct on the previous trial. The maximum correct delay was the maximum time of the delay imposed that resulted in a correct trial. A-not-B flag meant that at least one of the following conditions was present during the trial: (a) the tester uses only one well for the trial (instituted after the child missed the five previous trials); (b) the child uncovers both the wells at once; and (c) child partially reaches to uncover the well, and/or self-corrects when attempting to find the hidden toy.

The field workers performed the procedures for measuring anthropometry, drawing blood, and assessing socioeconomic status on a different day other than the cognitive tests. They completed daily quality-control assessments following standardized procedures to test the reliability of the equipment during this phase of data-collection. Weight was measured to the nearest 0.1 kg using a SECA floor scale (Seca Corporation, Hanover, Maryland, USA). Recumbent length was measured in triplicate to the 0.01 cm using a Shorr Board for length (Shorr Productions, Olney, Maryland). Three drops of blood were collected from each child using a heel prick. The first drop was wiped away, and the second and third drops were used for testing haemoglobin and erythrocyte protoporphyrin (EP) respectively using a haemoglobin photometer (HemoCue AB, Andelholm, Sweden) and a Hematoflurometer (AVIV Biomedical Inc., New Jersey, USA). The child's house was assessed for construction materials, water source, and the presence of material assets. Basic demographic information, including the child's sex, caste/religion, and birth date, was also documented from the caregiver's reports. When the caregivers were unable to remember the exact month and day of the child's birth, local calendars with the lunar cycle and a list of local festivals were used as aids. A timeline of the study activities is presented in Figure 2 .

### Randomization and supplementation

In the larger 2×2 factorial trial, the unit of randomization was a geographic sector, with several sectors comprising a VDC. Randomization was stratified by the VDC. Randomization for the 23 sectors within Ishwarpur VDC was carried out in 6 blocks of 4. The code assignments (A, B, C, or D) were determined from repeated drawing from a pool of all possible letter combinations. Hence, randomization was preserved within this selected VDC. All children living in a given sector were given the same supplement, facilitating the distribution of appropriate supplements by the field workers. Neither the families of the participants nor the research team were able to trace the code assignments to the supplements.

Children received: 6.25 mg of iron sulphate and 25 µg of folic acid, 5 mg of zinc sulphate, both iron sulphate-folic acid and zinc sulphate, or placebo; these doses correspond to half of a typical dose given to children aged over 12 months. The duration of supplementation for children who completed the 39-week assessments ranged from 0 to 24 weeks, and for those completing the 52-week visit, supplementation lasted from 0 to 37 weeks. Children consumed 0 to 237 supplements before receiving the information-processing tests. This variable duration was dictated by the need to test infants in the sub-study at the precise ages of 39 and 52 weeks. All children aged 1-35 months at the start of the larger trial were eligible for that trial.

### Analysis of data

Data were analyzed using the SPSS software (version 14.0) and the Stata software (version 8.0). The 39-and 52-week test data were analyzed separately. The preferences for novelty and fixation duration were extracted from the FTII program using the SAS software. The preference for novelty was described by the raw FTII score. A-not-B error, percentage of accurate performance, and percentage of deteriorated performance from the A-not-B Task are defined above.

The z-scores were calculated for three of the anthropometric measures using the 1978 Centers for Disease Control and Prevention (CDC)/World Health Organization (WHO) growth reference in the Epi Info software (version 6.0) (CDC, Atlanta, GA) to facilitate comparison with previous studies published using data from the same population (4,32-34). Stunting, wasting, and underweight were defined respectively as height-for-age (LAZ), weight-for-length (WLZ), and weight-for-age (WAZ) <-2 z-scores.

Principal component analysis was used for creating a socioeconomic status (SES) scale (35) from the questions on material assets and home amenities. The most comprehensive factor with the largest eigen value of >1 was selected. Twelve of the 17 related items were retained for the SES scale: presence of a latrine, servant, cattle, bicycle, radio, farmable land, home-garden plot, second floor on the house, roof material, television, electricity inside the house, and cart. Chronbach's alpha (36) of 0.78 was obtained, indicating good internal consistency. A higher score on the SES scale reflected a greater number of possessions. For regression models, the SES variable was dichotomized at the median, creating respectively low and high SES groups.

Fixation duration was log-transformed to create normal distributions. The significance was set at p<0.05 for the covariates to be entered and retained in the final model. Means of continuous variables and proportions of categorical variables were analyzed using *t*-and chi-square tests.

We hypothesized that the study children enrolled in the treatment group compared to the placebo group would have a higher percentage of novelty scores, shorter fixation-duration times, fewer A-not-B errors, a greater percentage of accurate performance, and less deteriorated performance. To test this, we calculated means and medians for the information-processing outcomes to test the potential differences between the treatment groups. The data were analyzed using the 2×2 factorial design and were then tested for the individual group differences for each of the outcomes. After finding no differences between the two methods, we chose to present the individual group differences for clarity. We created intention-to-treat linear and logistic models for each outcome with adjustment for cluster randomization only and then with adjustment for additional covariates by which the treatment groups differed before supplementation. Cluster randomization was achieved using generalized estimating equations (GEE) (37).

## RESULTS

The number of children who were eligible for the information-processing tests, later attempted and completed the FTII and A-not-B Task at 39 and 52 weeks, is presented in Figure 1. Descriptive characteristics, including sex, caste, SES, anaemia, IDA, and anthropometry did not differ (p>0.05) between children who completed and did not complete the tests (data not shown). However, when cluster randomization was controlled in a logistic regression model, the likelihood of females completing the A-not-B Task was lower than it was for males [odds ratio (OR)=0.67, 95% confidence interval (CI) 0.46-0.97, p<0.05]. When the supplementation groups were added to the above model, children who were enrolled in the supplementation groups were not found to differ from the children enrolled in the placebo group with respect to completing the A-not-B Task (data not shown).

Before the supplementation, 55% of the study infants were anaemic [haemoglobin (Hb) <105 g/L], 44% had IDA (Hb <105; erythrocyte protoporphyrin >90 µmol/mole heme), 22% were stunted [LAZ <-2 standard deviation (SD)], 13% were wasted (WLZ <-2 SD), and 42% were underweight (WAZ <-2 SD).

Despite randomization stratified at the VDC level, the four treatment groups differed before supplementation (Table 1). The zinc group was younger (52 weeks) and had more girls (39 weeks), families with a greater number of possessions (SES) (39 and 52 weeks), higher Hb values (39 and 52 weeks), and lower prevalence of anaemia and iron deficiency (39 and 52 weeks) (all p<0.05, Table 1). Children consumed supplements, on average, 5-7 days per week once supplementation began. No interactions between iron and zinc were found. Supplement dose was not found to be a significant predictor of performance in any of the models when entered either as a continuous or as a categorical variable (dichotomized as greater than or less than two months of supplementation before the test).

### The FTII

Compared to the 39-week infants, the 52-week infants had better mean FTII scores; they were one point higher on preference for novelty and 0.05 seconds faster on fixation duration (Table 2). In linear regression models adjusted for cluster randomization, the zinc-iron-folic acid group compared to the placebo group scored lower on preference for novelty at 52 weeks (Table 3). Lower preference for novelty in this group, compared to placebo at 52 weeks, remained significant after the models were further adjusted for sex, caste, and the number of supplements received (Table 4).

### The A-not-B Task

The 52-week-old infants scored better than the 39-week-old infants on two of the three A-not-B Task measures (Table 2). Their mean accurate performance score was six percentage points higher, and their deteriorated performance score was seven percentage points lower than the 39-week infants. However, despite having the same delay, the 52-week-old infants demonstrated more A-not-B errors than the 39-week-old infants (Table 2). In logistic models that controlled for cluster randomization only, the zinc-iron-folic acid group had higher likelihood of accurate (OR=2.94, CI 1.47-5.91) and lower likelihood of deteriorated (OR=0.35, CI 0.15-0.84) performance at 39 weeks compared to the placebo. However, at 52 weeks, this group had 84% higher odds (CI 1.04-3.26) of committing the A-not-B error than the placebo (p<0.05) (Table 3).

Multiple logistic regression models adjusted for cluster randomization, gender, caste, A-not-B flag, maximum delay, and the number of supplements received before the test revealed that, at 39 weeks, children receiving zinc-iron-folic acid had lower odds of both accurate and deteriorated performance than children who received a placebo. At 52 weeks, the zinc-iron-folic acid group compared to the placebo group had lower odds of accurate performance and of committing the A-not-B error (Table 4).

**Table 1.** Characteristics of infants randomized to treatment groups

Characteristics	39-week visit			
	Zinc	Iron/folic acid	Zinc/iron/folic acid	Placebo
Age (months) at enrollment^1^	7.1 (2.1)	7.5 (2.0)	7.1 (2.3)	7.1 (2.4)
% of females*	57.8	40.3	34.3	50
% of low-caste Hindus	75.8	87.1	75.8	60.7
% of Muslims	4.7	9.7	15.7	30.6
% of low SES score, below median^2^**	22.0	57.1	40.3	40.3
% of stunted infants^3^	14.9	10.3	15.3	26.3
% of wasted infants^4^	14.9	6.7	12.9	1.8
% of underweight infants^5^	26.1	31.0	36.5	36.8
Haemoglobin^1^***	110.3 (10.2)	100.6 (12.0)	102.2 (14.6)	102.0 (11.1)
% of haemoglobin <105^6^**	25.5	61.7	47.7	57.5
EP^1^*	81.7 (29.2)	129.1 (76.4)	122.8 (85.8)	108.0 (94.6)
% of EP >90^7^**	29.8	66.7	55.4	45.8
No. of supplements received^1^	29.4 (28.0)	26.6 (27.7)	33.4 (32.7)	25.6 (29.4)
	52-week visit			
Age (months) at enrollment^1^*	7.9 (2.7)	9.1 (2.8)	8.7 (3.0)	8.4 (3.0)
% of females	57.0	39.6	44.0	52.9
% of low-caste Hindus***	75.3	88.4	80.0	67.1
% of Muslims***	6.3	8.3	16.0	27.6
% of low SES score, below median^2^***	25.7	54.7	48.5	42.4
% of stunted infants^3^	14.5	19.3	25.9	25.0
% of wasted infants^4^	21.8	11.4	10.1	12.5
% of underweight infants^5^	32.1	40.4	44.0	48.7
Haemoglobin^1^***	109.1 (10.5)	101.1 (9.7)	102.0 (13.1)	101.5 (10.3)
% of haemoglobin <105^6^**	32.7	65.2	53.2	56.8
EP^1^**	89.4 (57.0)	130.3 (74.5)	129.8 (82.0)	114.5 (89.2)
% of EP >90^7^***	34.5	68.5	62.8	54.9
No. of supplements received^1^	67.0 (53.9)	50.3 (53.5)	58.9 (60.5)	54.2 (55.5)

*p<0.05;

**p<0.01;

***p<0.001;

^1^Values shown as mean (standard deviation)

^2^Number of assets; median=4 possessions;

^3^Length-for-age z-score <-2 standard deviations;

^4^Weight-for-length z-score <-2 standard deviations;

^5^Weight-for-age z-score <-2 standard deviations;

^6^Units given in g/L;

^7^Units given in µmol/mole heme;

EP=Erythrocyte protoporphyrin;

SES=Socioeconomic status

## DISCUSSION

We examined the effect of micronutrient supplementation at two time-points on five indicators of information processing in infants and found that zinc-iron-folic acid supplementation was associated with lower accuracy of performance at both the time-points, less deteriorated performance at 39 weeks, and fewer A-not-B errors at 52 weeks when the performance was adjusted for unbalanced randomization. Neither the combined nor the individual micronutrient supplements improved the performance on the FTII or the A-not-B Task.

Although we were able to administer both the tests in our remote field setting, the FTII required less effort from the child and was easier than the A-not-B Task to administer. We aligned the administration of both the tasks to coincide with the FTII eligibility because the FTII had a narrower window of eligibility than the A-not-B Task. However, we expected that some children received the FTII tests earlier than indicated, since it is possible that their true gestational age was shorter than the 40 weeks we assumed. In this setting, accurate gestational age is difficult to determine due to the rural and impoverished setting; neither the date of the last menstrual period (LPM) nor the true gestation age may be established. Given the study design, the pertinent comparison of the scores was between the supplemented group and the placebo group. The Nepali children's preference for novelty scores on the FTII were comparable with those measured in a Chilean sample of one-year old children (Nepal: 61.04 vs Chile: 61.9) (38).

**Table 2.** Descriptive statistics for 39-and 52-week testing periods

Test	39-week visit				
	Variable	No.	Mean (SD)/No. (%)	Minimum	Maximum
FTII	Preference for novelty	244	60.05 (6.16)	38.1	74.3
FTII	Fixation duration	233	0.319 (0.229)	-0.2	1.1
A-not-B Task	A-not-B error	202	48 (23.8)		
A-not-B Task	Accurate performance	200	74 (37)		
A-not-B Task	Deteriorated performance	200	43 (21.5)		
A-not-B Task	Neither accurate nor deteriorated	200	83 (41.5)		
	52-week visit				
FTII	Preference for novelty	325	61.0 (6.0)	40.8	78.2
FTII	Fixation duration	323	0.271 (0.18)	-0.14	0.95
A-not-B Task	A-not-B error	264	71 (26.9)		
A-not-B Task	Accurate performance	256	111 (43.4)		
A-not-B Task	Deteriorated performance	256	37 (14.5)		
A-not-B Task	Neither accurate nor deteriorated	256	108 (42.2)		

Preference for novelty: Raw FTII score. Fixation duration: Time spent attending to the stimuli. A-not-B error: No more than 1 error on repeat-following-correct trial, at least 1 error on a reversal trial and either an error on at least one more reversal trial or an error on the trial immediately following the reversal error. Accurate performance: ≥85% correct. Deteriorated performance: <75% correct;

>1 error on repeat-following-correct trials. Repeat-following-correct: Side of hiding remains same, and the subject was correct on previous trial;

FTII=Fagan Test of Infant Intelligence; SD=Standard deviation

The A-not-B Task was more difficult for the Nepali children to comprehend than the Fagan Test as many displayed signs of fatigue and stopped responding before the test was complete. Although many went on to complete the task, it is possible that the power was compromised. There is no standard protocol for administering the A-not-B Task, which makes cross-study comparisons very difficult. Testers who use this task impose a delay that is typically increased with age to ensure that the child is continually challenged (34). Despite having the same delay for the 39-and 52-week old infants in the Nepal study, the older children had a larger percentage of A-not-B errors, which is surprising and indicates possible problems for the older children. Nevertheless, the 52-week old infants appeared to benefit from zinc-iron-folic acid supplementation in having a lower likelihood of committing the A-not-B error.

The finding that males were more likely to complete the A-not-B Task than females mirrors recent findings on spatial ability that have shown gender differences as early as infancy (39,40). Spatial ability was required of the children to perform the A-not-B Task; however, the effect disappeared once the data were adjusted for caste and socioeconomic status. The sex differences that were revealed when the data were adjusted for cluster randomization but not when caste and socioeconomic status were adjusted can be attributed to confounding.

Many factors potentially affect the relationship between the nutritional status and the poor developmental outcomes. These include low SES (41,42), lack of stimulation in the home (43), poor maternal education (44,45), low birthweight (45), and lower rates of breastfeeding and early weaning (9). We differentiated between the low and the high SES; however, the differences were not large. Our SES measure revealed that few material resources were available in this population. Furthermore, only 25% of women living in the study area were reported to be literate (3,26). Birthweight was not measured in this study. However, based on previous research, we expect that a large percentage of our study children had low birthweight (46,47). Preliminary research revealed that 95% of our study subjects were breastfeeding and that few complementary foods were introduced during the first year of life (4,32). In this impoverished environment where the study participants were living in the presence of an ongoing civil insurgency and other risk factors, low-level supplementation of micronutrients may not have been sufficient to improve information processing in these infants.

**Table 3.** Effect of supplementation on mean FTII scores and A-not-B Task odds ratios at 39 and 52 weeks of age from multivariate models adjusted for cluster randomization

Test	39-week visit				
	Outcome	Placebo OR (95% CI)	Zinc OR (95% CI)	Iron-folic acid OR (95% CI)	Zinc-iron-folic acid OR (95% CI)
FTII	Preference for novelty^1^	60.76 (59.51-60.01)	58.76 (56.80-60.72)	60.80 (59.46-62.15)	60.08 (58.21-61.94)
FTII	Fixation duration^2^	1.40 (1.26-1.56)	1.39 (1.33-1.46)	1.30 (1.26-1.35)	1.41 (1.33-1.49)
A-not-B Task	A-not-B error^3^	1.0	0.81 (0.38-1.74)	0.67 (0.17-2.57)	1.42 (0.54-3.73)
A-not-B Task	Accurate performance^4^	1.0	1.31 (0.52-3.27)	1.11 (0.48-2.55)	2.94 (1.47-5.91)6
A-not-B Task	Deteriorated performance^5^	1.0	0.66 (0.29-1.52)	1.37 (0.52-3.58)	0.35 (0.15-0.84)6
	52-week visit				
FTII	Preference for novelty^1^	62.06 (60.98-63.14)	61.37 (58.46-64.28)	61.40 (60.21-62.58)	60.0 (59.03-60.97)6
FTII	Fixation duration^2^	1.30 (1.27-1.36)	1.31 (1.27-1.36)	1.30 (1.28-1.33)	1.32 (1.28-1.37)6
A-not-B Task	A-not-B error^3^	1.0	1.47 (0.80-2.69)	1.38 (0.93-2.04)	1.84 (1.04-3.26)6
A-not-B Task	Accurate performance^4^	1.0	0.89 (0.50-1.60)	0.91 (0.50-1.70)	0.83 (0.50-1.36)
A-not-B Task	Deteriorated performance^5^	1.0	1.06 (0.58-1.94)	0.98 (0.48-1.99)	0.88 (0.34-2.26)

^1^Preference for novelty: Raw FTII score;

^2^Fixation duration: Time spent attending to the stimuli;

^3^A-not-B error: No more than 1 error on repeat-following-correct trial—at least 1 error on a reversal trial and either an error on at least one more reversal trial or an error on the trial immediately following the reversal error. Repeat-following-correct: Side of hiding remains same and subject was correct on previous trial;

^4^Accurate performance: ≥85% correct;

^5^Deteriorated performance: <75% correct;

>1 error on repeat-following-correct trials;

6p<0.05;

CI=Confidence interval;

FTII=Fagan Test of Infant Intelligence;

OR=Odds ratio

Other limitations that could have affected the results of the study include the selection of a purposive sub-sample from a large cluster randomized trial, the choice to randomize at the sector level and analyze the data at the individual level, and the variation in supplement dose that occurred when trying to administer the cognitive testing within the methodological framework of a larger study. We controlled for cluster randomization in each of the models, used an intent-to-treat analysis, and further controlled for the baseline characteristics that differed among the treatment groups, despite the randomization and supplement dose. Options for working within the context of a developing country are often limited. However, in light of the paucity of data on specific cognitive functions in very young micronutrient-deficient children, our study provides important findings on measures of information processing.

Few other researchers have used the information-processing outcomes to measure the cognitive differences in young children supplemented with zinc and iron. In fact, as far as we know, there are no studies on these outcomes in zinc-supplemented individuals. Research conducted with Mexican-American school children and men found that zinc had an impact on attention and memory (48). With respect to iron, a non-randomized study among Chilean full-term infants examined the effects of no-added iron, low-or high-added iron on the FTII and found a positive effect of iron supplementation compared to the ‘no-added iron’ group on fixation duration (36) but not on preference for novelty. Chilean infants supplemented with high versus low-iron for six months had shorter fixation duration (38). In comparison, the participants in this study were randomized, and the study itself was conducted in a more impoverished environment. Nepal is classified as one of the least-developed and poorest nations in the world (49).

**Table 4.** Effect of supplementation on mean FTII scores and A-not-B Task odds ratios at 39-and 52-weeks of age from multivariate models adjusted for cluster randomization-sex-caste-and dose^1^

Test	39-week visit				
	Outcome	Placebo OR (95% CI)	Zinc OR (95% CI)	Iron-folic acid OR (95% CI)	Zinc-iron-folic acid OR (95% CI)
FTII	Preference for novelty^2^	59.38 (57.52-61.25)	57.46 (55.47-59.44)	59.41 (57.65-61.17)	58.63 (55.79-61.47)
FTII	Fixation duration^3^	1.42 (1.29-1.57)	1.40 (1.35-1.53)	1.32 (1.25-1.38)	1.46 (1.36-1.57)
A-not-B Task	A-not-B error^4^	1.0	0.33 (0.20-0.56)	0.31 (0.09-1.06)	0.70 (0.34-1.41)
A-not-B Task	Accurate performance^5^	1.0	0.18 (0.06-0.56)	0.17 (0.07-0.41)	0.38 (0.20-0.71)^7^
A-not-B Task	Deteriorated performance^6^	1.0	0.41 (0.23-0.75)	0.95 (0.38-2.38)	0.24 (0.11-0.56)^7^
	52-week visit				
FTII	Preference for novelty^2^	62.10 (60.86-63.35)	61.41 (58.02-64.79)	61.48 (60.04-62.91)	60.10 (58.84-61.36)^7^
FTII	Fixation duration^3^	1.30 (1.26-1.34)	1.33 (1.27-1.39)	1.30 (1.27-1.34)	1.33 (1.28-1.39)
A-not-B Task	A-not-B error^4^	1.0	0.30 (0.13-0.70)	0.40 (0.25-0.64)	0.48 (0.28-0.83)^7^
A-not-B Task	Accurate performance^5^	1.0	0.59 (0.29-1.19)	0.48 (0.21-1.09)	0.37 (0.22-0.62)^7^
A-not-B Task	Deteriorated performance^6^	1.0	0.16 (0.78-0.34)	0.16 (0.07-0.40)	0.14 (0.05-0.43)

^1^A-not-B Task outcomes were additionally adjusted for A-not-B flag and maximum delay. Dose and maximum delay were centred around their respective means;

^2^Preference for novelty: Raw FTII score;

^3^Fixation duration: Time spent attending to the stimuli;

^4^A-not-B error: No more than 1 error on repeat-following-correct trial—at least 1 error on a reversal trial and either an error on at least one more reversal trial or an error on the trial immediately following the reversal error. Repeat-following-correct: Side of hiding remains same, and subject was correct on previous trial;

^5^Accurate performance: ≥85% correct;

^6^Deteriorated performance: <75% correct; >1 error on repeat-following-correct trials;

^7^p<0.05;

CI=Confidence interval;

FTII=Fagan Test of Infant Intelligence;

OR=Odds ratio

The use of randomized, placebo-controlled clinical trials and specific information-processing tests are keys to understanding the associations between micronutrient status and cognitive function in young preverbal children. The primary strengths of this study were the use of two specific measures of information processing—the FTII and the A-not-B-Task, as opposed to the more commonly-used sensorimotor tests of infant development. This study evaluated the information processing of young children during the first year of their life in a developing-country setting where cognitive development is rarely investigated. A randomized trial design was used for reducing the likelihood of bias; however, there were still some differences by treatment group at baseline. These were adjusted for in the final analysis. It is possible that there may be some residual bias from factors not adjusted for but this is not likely to fully explain the results, since both randomization and adjustment were applied to the design and analysis. In sum, the evidence from this experimental trial, coupled with the evidence from previous observational studies with heterogeneous development outcomes, revealed little consistency in the effect of iron and/or zinc supplementation on indicators of infant cognition.

### Conclusions

These findings point to the need to elucidate the role of micronutrients in early cognitive development and further explore the influence of gender on specific cognitive indicators. Future studies with fewer limitations will need to be designed to further investigate the effect of micronutrient supplementation on the information-processing outcomes among young micronutrient-deficient children. Moreover, future research needs to address the broader contextual question of early cognitive development as children are frequently exposed to a host of factors that are not illuminated as part of the research design. In environments with significant stressors, such as the stress of the ongoing civil insurgency that occurred throughout this study, supplementation with nutrients, such as magnesium and vitamin C, that are depleted from tissues and poorly absorbed when the body is engaged in sympathetic versus parasympathetic nervous activity (50-52), may have a more pronounced effect on early brain development than iron and zinc. Results of recent human and animal studies provide evidence for negative effects of vitamin C and magnesium deficiencies on neonatal brain functioning (53-55). Perhaps future supplementation trials aimed at impacting early cognitive development need to account for nutrient depletion due to the stress response.

## ACKNOWLEDGEMENTS

The study was funded by the National Institutes of Health (HD 38753), the Bill & Melinda Gates Foundation (810-2054), and a Cooperative Agreement between the Johns Hopkins University and the Office of Health and Nutrition, U.S. Agency for International Development (HRN-A_00-97-00015-00).
